# How molecular techniques are developed from natural systems

**DOI:** 10.1093/genetics/iyad067

**Published:** 2023-05-16

**Authors:** Isobel Ronai

**Affiliations:** Charles Perkins Centre and School of Life and Environmental Sciences, The University of Sydney, Sydney 2006, Australia; Department of Organismic and Evolutionary Biology, Harvard University

**Keywords:** RNAi, PCR, GFP, iPS, CRISPR-Cas, gene knockdown, gene silencing, co-suppression, philosophy of biology, scientific practice

## Abstract

A striking characteristic of the molecular techniques of genetics is that they are derived from natural occurring systems. RNA interference, for example, utilizes a mechanism that evolved in eukaryotes to destroy foreign nucleic acid. Other case studies I highlight are restriction enzymes, DNA sequencing, polymerase chain reaction, gene targeting, fluorescent proteins (such as, green fluorescent protein), induced pluripotent stem cells, and clustered regularly interspaced short palindromic repeats-CRISPR associated 9. The natural systems’ strategy for technique development means that biologists utilize the activity of a mechanism's effector (protein or RNA) and exploit biological specificity (protein or nucleic acid can cause precise reactions). I also argue that the developmental trajectory of novel molecular techniques, such as RNA interference, has 4 characteristic phases. The first phase is discovery of a biological phenomenon. The second phase is identification of the biological mechanism's trigger(s): the effector and biological specificity. The third phase is the application of the trigger(s) as a technique. The final phase is the maturation and refinement of the technique. Developing new molecular techniques from nature is crucial for future genetic research.

## Introduction

Biologists can explain the complex phenomena underlying living processes by identifying the genetic mechanisms that produce such processes (Schaffner 1996; Darden 2006; Tabery *et al*. 2015). To access the causal structure of genetic mechanisms, biologists use sophisticated molecular techniques to manipulate the components of the mechanism and observe the resulting effects. Scientific knowledge in genetics therefore progresses in a distinctive way; progress is driven by the introduction and use of novel techniques (Vance 1996; Waters 2008). In contrast, ecology is an area of biology that has progressed through theoretical developments and model building (Sarkar and Elliott-Graves 2016). What drives the development of molecular techniques in genetics?

### From natural systems to techniques

A striking feature of the development of molecular techniques, which biologists themselves often highlight (for example, Mello and Conte 2004; Lander 2016), is that they are derived from naturally occurring systems. These techniques are not developed through “rational design” using engineering principles (discussed in O’Malley 2009), do not utilize physiochemical properties (such as microscopy and gel electrophoresis), nor do they mimic nature (Ahn *et al*. 2015).

I will highlight 8 contemporary molecular techniques that are derived from natural systems, as highly successful and prominent examples. These techniques have been patented, led to landmark scientific articles, and been the subject of a Nobel Prize (Ronai and Griffiths 2019). Therefore, the scientific community sees these 8 techniques as significant advances. In chronological order, these techniques are restriction enzymes, DNA sequencing, polymerase chain reaction (PCR), gene targeting, fluorescent proteins (such as, green fluorescent protein), RNA interference (RNAi), induced pluripotent stem cells (iPS), and clustered regularly interspaced short palindromic repeats-CRISPR associated 9 (CRISPR-Cas9) (see Table 1 for a description of the techniques). These 8 molecular techniques are so ubiquitous that they are regarded as common knowledge by geneticists, and when these techniques are mentioned in the Methods section of a scientific article, a citation for the technique is not required (see for example RNAi in Ronai *et al*. 2016).

**Table 1.** Summary and characterization of 8 highly successful molecular techniques (in chronological order of development).

Technique	Description	Originating natural system	Biological function of mechanism	Experimental context	Type of effector	Type of biological specificity
**Restriction enzymes**	Introduction of restriction enzyme to cut DNA into specified fragments	Bacteria (*Haemophilus influenzae*)	Destroy foreign nucleic acid from bacteriophages	In vitro	Exogenous restriction endonuclease	Stereochemical: DNA recognition sequence
**DNA sequencing**	Synthesis of DNA to determine the sequence of bases (A, T, C and G)	Bacteria (*Escherichia coli*)	DNA replication	In vitro	Exogenous DNA polymerase I	Informational: dideoxynucleotides (also DNA primer) has sequence match to DNA template
**PCR**	Synthesis of DNA to amplify a specified region	Bacteria (*E. coli*)	DNA replication	In vitro	Exogenous DNA polymerase I	Informational: DNA primers have sequence match to DNA template
**Gene targeting**	Introduction of homologous DNA fragments to replace region of DNA in experimental system	Organism or cell culture (*Homo sapiens*)	Homologous recombination	Organisms & cell culture	Endogenous endonuclease (for example, SPO11)	Informational: exogenous DNA has sequence match to target DNA/gene
**Fluorescent proteins**	Introduction of fluorescent proteins to track biological processes in experimental system	Jellyfish (*Aequorea victoria*)	Unknown—emitted when jellyfish is agitated (Davenport and Nicol 1955)	Organisms & cell culture	Exogenous fluorescent protein, in particular the fluorophore	Engineered informational specificity: fluorescent protein DNA placed in specific location
**RNAi**	Introduction of RNA to reduce gene expression in experimental system	Eukaryote (*Caenorhabditis elegans*) or cell culture	Destroy foreign nucleic acid or gene regulation	Eukaryote organisms & cell culture	Endogenous RISC complex, in particular the Argonaute endonuclease	Informational: dsRNA (& siRNA) with sequence match to target mRNA
**iPS**	Introduction of transcription factors (Yamanaka factors) reprograms somatic cells to become pluripotent	Embryonic stem cells (*Mus musculus*)	Stem cell function (unlimited self-renewal & pluripotency)	Cell culture	Exogenous transcription factors (*Oct4*, *Sox2*, *cMyc*, & *Klf4*)	Stereochemical: DNA binding site
**CRISPR-Cas9**	Introduction of Cas protein and single-guide RNA to edit DNA in experimental system	Bacteria (*Streptococcus pyogenes*)	Destroy foreign nucleic acid from bacteriophages	Organisms & cell culture	Exogenous RNA-guided DNA endonuclease (Cas9)	Informational: guide RNA (crRNA + tracrRNA) with sequence match to target DNA

These techniques are all derived from natural systems and are now utilized as methodologies. For key references and timeline, see Table 2.

The 8 highly successful molecular techniques examined are derived from mechanisms that each evolved for a particular biological function in a natural system (see Table 1). The biological function of the RNAi mechanism, for example, is a eukaryotic defense system for the destruction of foreign nucleic acid and mobile elements (Waterhouse *et al*. 1998, 2001; van Rij and Andino 2006). In addition, the RNAi mechanism is thought to have been repurposed (Cerutti and Casas-Mollano 2006) for the precise regulation of endogenous gene expression, in particular for the regulation of developmental genes (Carrington and Ambros 2003). The same biological function, to destroy foreign nucleic acid in an organism, underlies the techniques of RNAi (derived from eukaryotes) and CRISPR-Cas9 (derived from prokaryotes) (Bhaya *et al*. 2011; Wright *et al*. 2016), but the 2 techniques involve different molecular mechanisms (Table 1). Therefore, the “arms race” that occurs between viruses and their organismal hosts has provided biologists with the basis of 2 techniques.

Natural systems show biologists what is mechanistically possible and natural mechanisms have been selected by evolution so are likely to have a high level of effectiveness (Arnold 2018). However, the components of these natural mechanisms are contingent on historical, iterative events rather than being at an optimal state. Biologists can alter these components to reach an optimal state but are constrained by their possibility space (Arnold 2015).

Biologists use molecular techniques developed from preexisting, natural mechanisms because they are compatible with living processes (Weber 2017) and do not create artificial phenomena. Furthermore, the use of a natural mechanism may allow the continuing function of the biological process (for example, fluorescent proteins), and cellular-based techniques can be stably inherited in designed constructs with transgenerational effects (Chalfie *et al*. 1994). These techniques can therefore be used to observe or intervene in active, complex biological processes even when no comprehensive understanding of these processes exists.

### The importance of natural systems for the development of molecular techniques

I propose that molecular techniques are developed by exploiting 2 key components of natural mechanisms: an effector molecule's (such as, proteins or RNAs) activity and the use of biological specificity (protein or nucleic acid can cause precise reactions). The importance of an effector's activity and the use of biological specificity for molecular techniques are often implicitly recognized by geneticists. For example, many studies on RNAi highlight the technique's effector, which is the RNA-induced silencing complex (RISC) (see Vaucheret *et al*. 1998; Filipowicz *et al*. 2005; Li *et al*. 2006; Rana 2007; Siomi and Siomi 2009; Fellmann and Lowe 2014), and that specificity is derived from double-stranded RNA (dsRNA) (see Fire *et al*. 1998; Kennerdell and Carthew 1998; Waterhouse *et al*. 1998; Hamilton and Baulcombe 1999; Hammond *et al*. 2000; Parrish *et al*. 2000; Hammond *et al*. 2001; Waterhouse *et al*. 2001; Elbashir *et al*. 2001b; Bartel 2004; Rana 2007; Siomi and Siomi 2009; Fellmann and Lowe 2014).

### Effector activity

Living systems use effector molecules to generate a particular activity within a mechanism (Bich and Bechtel 2022). I have identified the protein effector, all from a natural system, for each of the 8 highly successful molecular techniques (Table 1). The majority of the techniques utilize proteins that are catalytic enzymes, and the techniques leverage the efficiency of the enzymatic activity (Table 1). The 2 exceptions are the techniques of fluorescent proteins and iPS which utilize a protein's stereochemistry, a fluorophore or structural motif, respectively (Table 1).

A technique's effector is either endogenous or exogenous to the experimental system (Table 1). Endogenous effector techniques use the effector for its original purpose but appropriate the overall mechanism. For example, the effector of RNAi is the RISC, which is an endogenous component of a molecular mechanism present in all eukaryotes (Cerutti and Casas-Mollano 2006). Exogenous effector techniques use the effector for its original biological function, but in another biological context. For example, the effector of a restriction enzyme experiment is a restriction endonuclease, which is a component that must be added to the experiment. As the exogenous effector is introduced into the experimental system (either permanently or transiently), it is more tractable than an endogenous effector.

### Biological specificity

Living systems need biological specificity to achieve precise control over their molecular mechanisms (Woodward 2010; Griffiths *et al*. 2015). In the 8 highly successful molecular techniques examined, geneticists introduce biological specificity into their experimental systems to precisely access the target mechanism with fine-grained control (Waters 2007). Geneticists need interventions with minimal off-target events. Also, high specificity means that the technique can be “multiplexed,” as multiple nucleic acid sites can be targeted at the same time. I have identified that the majority of the 8 molecular techniques use nucleic acid sequence informational specificity (Griffiths and Stotz 2013); nucleic acid is the substrate of the mechanism (Table 1). For example, RNAi provides fine-grained control of gene expression because it uses nucleic acid sequence informational specificity. Before RNAi, such modulation of gene expression was not possible (Bellés 2010). One molecular technique, fluorescent proteins, uses what I term “engineered informational specificity,” where the geneticist creates the specificity by placing the effector in a highly specific location. The last 2 molecular techniques, iPS and restriction enzymes (Table 1), use protein stereochemical specificity (Griffiths and Stotz 2013). For techniques that have stereochemical specificity (Table 1), the effector provides the specificity.

### The importance of an effector's activity and biological specificity

If there are multiple techniques available to achieve the same experimental purpose, then the technique with the greatest efficiency or superior type of specificity is preferred by the scientific community. For example, 3 recent techniques used for the purpose of DNA editing are zinc finger nucleases (ZFNs), a technique that uses 2 protein domains coupled together (Kim *et al*. 1996); transcription activator-like effector nucleases (TALENs), a technique derived from the bacteria *Xanthomonas* (Boch *et al*. 2009; Moscou and Bogdanove 2009); and CRISPR-Cas9 (Table 1). A ZFNs’ and TALENs’ specificity is stereochemical, so they require proteins to be reengineered for every experiment. These 2 techniques are therefore not as easily programmable for a wide range of targets when compared with CRISPR-Cas9, which uses a guide RNA (informational specificity). The superior specificity of CRISPR-Cas9 has meant that it has been commercially viable and has replaced ZFNs and TALENs as the premier genome-editing technique (Doudna and Charpentier 2014; Corbyn 2015). The effector activity and specificity of a technique are critical to its success.

### Molecular technique development has 4 phases

I propose that molecular techniques derived from natural systems have a specific pattern of historical development, with 4 critical phases. These phases are the discovery of a biological phenomenon, identification of the biological mechanism's trigger(s) (the specificity and effector components), application of the technique, and maturation of the technique. Each of the 8 highly successful molecular techniques shows the 4 phases of technique development (see Table 2). I use RNAi as a detailed case study due to its contemporary history. This technique introduces molecules of RNA into an organism or cell to reduce the expression of a gene of interest (reviewed in Mello and Conte 2004).

**Table 2.** The 4 phases of development for the 8 highly successful molecular technique case studies (in chronological order of development).

Technique	Phase	Reference	Description
**Restriction enzymes**	**1. Discovery**	Luria and Human (1952)	Discovered that bacteriophage (T1, T2, T3, T4, T5, T6, and T7) vary in their ability to grow in different bacterial (*Escherichia coli* and *Shigella dysenteriae*) strains.
		Dussoix and Arber (1962)	Discovered that bacteriophage λ DNA degrades in *E. coli* strains.
	**2. Identification of triggers (effector/specificity)**	Kelly Jr and Smith (1970), Smith and Welcox (1970) (study in 2 parts)	Identified the nucleotide recognition sequence that causes restriction enzymes (in particular, a type II which recognizes DNA and cuts sites at the same place, endonuclease R from *Haemophilus influenzae*) to cut DNA.
	**3. Application of trigger**	Danna and Nathans (1971)	Applied restriction enzyme (endonuclease R from *H. influenzae*) to cut up DNA.
	**4. Maturation**	Feinberg and Vogelstein (1983)	Developed restriction enzymes using radiolabeling to efficiently recover DNA fragments.
**DNA sequencing**	**1. Discovery**	Watson and Crick (1953)	Discovered the complementary DNA structure in calf thymus (possibly) and proposed a mechanism for DNA replication. Also, predicted the existence of DNA polymerase.
		Matthaei *et al*. (1962)	Discovered that 3 nucleotides code for a specific amino acid in a cell-free system of *E. coli*. Also, predicted the code was universal.
	**2. Identification of trigger (effector)**	Kornberg *et al*. (1956b)	Identified DNA polymerase in *E. coli*.
	**2. Identification of trigger (specificity)**	Atkinson *et al*. (1969)	Identified that dideoxynucleotides cause DNA polymerase to terminate synthesis of DNA.
	**3. Application of triggers**	Sanger *et al*. (1977)	Applied dideoxynucleotides with DNA polymerase from *E. coli* to determine the DNA sequence of bacteriophage φX174.
	**4. Maturation**	The *C. elegans* Sequencing Consortium (1998)	Developed DNA (Sanger) sequencing to sequence the first multicellular organism (*Caenorhabditis elegans*) genome.
		International Human Genome Sequencing Consortium (2001)	Developed DNA (Sanger) sequencing to sequence the human genome.
**PCR**	**1. Discovery**	Watson and Crick (1953)	Discovered the complementary DNA structure in calf thymus (possibly) and proposed a mechanism for DNA replication. Also, predicted the existence of DNA polymerase.
		Meselson and Stahl (1958)	Discovered that DNA replicates semi-conservatively in *E. coli*.
	**2. Identification of trigger (effector)**	Kornberg *et al*. (1956b)	Identified DNA polymerase in *E. coli*.
	**2. Identification of trigger (specificity)**	Kornberg *et al*. (1956a)	Identified that a primer causes DNA polymerase to initiate synthesis of DNA.
	**3. Application of triggers**	Saiki *et al*. (1985)	Applied primers with DNA polymerase from *E. coli* to amplify DNA region.
	**4. Maturation**	Saiki *et al*. (1988)	Developed PCR to be thermostable using DNA polymerase from *Thermus aquaticus*.
**Gene targeting**	**1. Discovery**	Gluzman *et al*. (1977); Vogel *et al*. (1977) (study in 2 parts)	Discovered that a mutant phenotype can be rescued in a simian virus 40 (SV40) temperature-sensitive mutant (tsD202) when added to monkey CV1 cells (containing endogenous integrated SV40). Also, discovered that the rescue is due to recombination.
	**2. Identification of trigger (specificity)**	Hinnen *et al*. (1978)	Identified that exogenous DNA of *LEU2* causes site-specific recombination with homologous chromosomal DNA in *Saccharomyces cerevisiae*.
	**2. Identification of trigger (effector)**	A single study cannot be identified because the biological mechanism underlying gene targeting has multiple effectors	Identified that an endogenous endonucleases create a double-stranded break and this initiates repair pathway. For example, SPO11.
	**3. Application of trigger**	Smithies *et al*. (1985)	Applied exogenous DNA to modify only the target gene (β-globin) in human cells.
	**4. Maturation**	Thomas and Capecchi (1987)	Developed gene targeting to inactivate an endogenous gene (*hptr*) in mouse embryonic stem cells.
		Doetschman *et al*. (1987)	Developed gene targeting to correct mutant *hptr* in mouse embryonic stem cells.
		Mansour *et al*. (1988)	Developed gene targeting selection (positive for cells that have incorporated exogenous DNA and negative for cells that have randomly incorporated exogenous DNA) in mouse embryonic stem cells.
**Fluorescent proteins**	**1. Discovery**	Davenport and Nicol (1955)	Discovered the green fluorescence in *Aequorea victoria*.
	**2. Identification of trigger (effector)**	Shimomura *et al*. (1962)	Identified the green fluorescent protein (GFP) in *Aequorea victoria*.
	**2. Identification of trigger (specificity)**	Prasher *et al*. (1992)	Identified the genomic DNA and cDNA sequence of GFP that causes fluorescence in *Aequorea victoria*.
	**3. Application of trigger**	Chalfie *et al*. (1994)	Applied GFP cDNA to generate fluorescence in *E. coli* and *Caenorhabditis elegans* cells.
	**4. Maturation**	Heim *et al*. (1995)	Developed GFP spectral characteristics using a point mutation in *E. coli*.
		Cormack *et al*. (1996)	Developed GFP variants that fluoresce at higher intensity in *E. coli*.
**RNAi**	**1. Discovery**	Napoli *et al*. (1990)	Discovered the knockdown of *chalcone synthase* in *P. hybrida*.
	**2. & 3. Identification of trigger (specificity) & application of trigger**	Fire *et al*. (1998)	Identified that dsRNA causes sequence specific regulation of mRNA in *Caenorhabditis elegans*. Applied dsRNA to knockdown gene expression in *Caenorhabditis elegans*.
	**2. Identification of trigger (specificity processed component)**	Hamilton and Baulcombe (1999)	Identified that siRNA (processed product of dsRNA) causes sequence specific regulation of mRNA in plants.
	**2. Identification of trigger (effector)**	Hammond *et al*. (2000)	Identified the endogenous RISC complex which contains an endonuclease that cleaves target mRNA in *Drosophila* cells.
	**4. Maturation**	Elbashir *et al*. (2001a)	Developed RNAi to knockdown gene expression in mammalian and *Drosophila* cells.
**iPS**	**1. Discovery**	Gurdon (1962)	Discovered that cell differentiation is reversible, as the nucleus of a somatic cell can successfully replace the nucleus of an egg cell in *Xenopus laevis*.
	**2. & 3. Identification of trigger (effector/specificity) & application of trigger**	Takahashi and Yamanaka (2006)	Identified the genome and transcriptome changes that cause 4 transcription factors (Oct3/4, Sox2, c-Myc, and Klf4 in mice) to make somatic cells become pluripotent stem cells. Applied the 4 transcription factors cDNA to reprogram embryonic and adult fibroblast mice cells.
	**4. Maturation**	Takahashi *et al*. (2007)	Developed iPS in human cells.
**CRISPR-Cas9**	**1. Discovery**	Ishino *et al*. (1987)	Discovered the CRISPR motif (repeated sequence with spacers) in the DNA sequence of *E. coli*.
	**2. Identification of effector**	Makarova *et al*. (2002)	Identified the CRISPR-associated (*cas*) genes in the genome sequences of Bacteria and Archaea. In particular, the class 2, Type II (recognizes DNA and cleavage results in double-stranded break) Cas9 (COG3513) in *Streptococcus pyogenes*, *Campylobacter jejuni*, *Neisseria meningitidis*, and *Pasteurella multocida*.
	**2. Identification of trigger (specificity component A)**	Brouns *et al*. (2008)	Identified that CRISPR RNAs (crRNAs) cause Cas9 to sequence specifically cleave DNA in *E. coli*.
	**2. & 3. Identification of trigger (specificity component B) & application of triggers**	Jinek *et al*. (2012)	Identified that crRNA and transactivating CRISPR RNA (tracrRNA) must complementary base pair to cause Cas9 to site-specifically cleave DNA. Applied a tracrRNA-crRNA complex (the “single-guide RNA”) with Cas9 from *S. pyogenes* to cleave DNA.
	**3. Application of triggers**	Gasiunas *et al*. (2012)	Applied crRNA with Cas9 from *Streptococcus thermophilus* to cleave DNA.
	**4. Maturation**	Cong *et al*. (2013)	Developed CRISPR-Cas9 to edit the genome of mammalian (human and mouse) cells.
		Mali *et al*. (2013)	

For each technique, I highlight the published papers, in chronological order, for each of the 4 phases: discovery of the biological phenomenon, identification of the biological mechanism's triggers, application of the trigger(s) as a technique, and examples of highly cited papers that demonstrate the maturation of the technique.

### The first phase: discovery of a biological phenomenon

In the first phase of technique development, biologists identify and describe an unusual phenomenon in a natural system. At this stage, the underlying mechanism is not well characterized, and the biological function of the mechanism is typically unknown. These studies can be identified by examining the studies that the later phases build upon.

For example, in the early 1990s, the RNAi phenomenon was first identified in plants (Table 2). Napoli *et al*. (1990) and van der Krol *et al*. (1990) aimed to increase color intensity in the *Petunia hybrida* flower and introduced synthetic sense RNA into the plant in order to overexpress a gene in the pathway that controls formation of the flower pigment. Contrary to expectation, these flowers had reduced pigment, rather than more. Therefore, the sense RNA had reduced the mRNA of the endogenous gene. During the 1990s, multiple studies were conducted on how different organisms actively respond to the introduction of RNA (Fire *et al*. 1991; Romano and Macino 1992; Guo and Kemphues 1995; Lin *et al*. 1995; Mello *et al*. 1996; Powell-Coffman *et al*. 1996; Guedes and Priess 1997). At this time, the RNAi phenomenon was described using many different terms: the initial study by Napoli *et al*. (1990) termed this phenomenon “co-suppression,” but a follow-up study (Van Blokland et al. 1994) demonstrated that silencing occurred posttranscriptionally, so the phenomenon was then referred to as “posttranscriptional gene silencing.” Another study identified the RNAi phenomenon in a fungus, *Neurospora crassa*, and termed it “quelling” (Romano and Macino 1992). While the term “RNA-mediated interference” was coined in an early *Caenorhabditis elegans* RNAi study (Rocheleau *et al*. 1997). These early studies on RNA produced knowledge that was critical to the development of RNAi.

### The second phase: identification of the trigger(s) of a biological mechanism

In the second phase of technique development, biologists identify the specificity and effector component of the mechanism (see Table 3a and b). I term the specificity and effector components of a mechanism as trigger(s) because they are the key causative agents and are the “causally specific actual difference maker” under typical conditions (Carrier 2004; Waters 2007; Woodward 2010). Once biologists identify the trigger(s), they can use it to precisely manipulate the mechanism. If the effector is endogenous to the experimental system (Table 1), then it does not need to be added to the experiment and its identification is not essential for the development of the technique. However, effectors that are exogenous to the experimental system are identified before the specificity trigger (Table 1).

**Table 3.** Key experiments for the RNAi technique conducted by Fire *et al*. (1998).

(a)		
Specificity	Range tested	Result
Nonpurified single-stranded RNA (ssRNA)	Sense RNA or antisense RNA	Introduction of nonpurified ssRNA into the experimental system caused RNAi.
Purified ssRNA	Sense RNA or antisense RNA	Purified ssRNA led to weaker RNAi compared with purified dsRNA, indicating that dsRNA causes RNAi.
Complementary sense and antisense strand RNA	Preannealed, injected sequentially, or injected sequentially but with long time interval between RNAs	Preannealing RNA led to stronger RNAi, indicating that the formation of dsRNA was important for RNAi. Sequential injection of sense and antisense RNA led to RNAi, indicating that RNA strands could hybridize to form dsRNA in an experimental system. A longtime interval between sequential injection of RNAs led to no RNAi, indicating that ssRNA are degraded or become inaccessible in the experimental system.
Time postinjection of RNA	6, 15, 27, 41, or 56 h	The longer the time interval after the introduction of RNA into the experimental system the effect of RNAi decreased, indicating RNAi relies on the introduction of RNA.
ssRNA and control gene dsRNA	ssRNA not attached to dsRNA, ssRNA attached at its 5′ end to dsRNA, or ssRNA attached at its 3′ end to dsRNA	ssRNA attached to dsRNA controls led to no RNAi, indicating that sequence specificity not a double-stranded structure was important for RNAi.
dsRNA length	299–1,033 nucleotides	Nucleotide length of dsRNA did not affect RNAi.
RNA dosage	30,000–3,600,000 RNA molecules per organism	Low dosages of dsRNA triggered RNAi, indicating that RNAi is a catalytic process (i.e. enzymes involved); otherwise, there would not be enough RNA molecules to bind to all of the endogenous mRNA in the experimental system.
Site of injection of RNA in organism	Body cavity of head, body cavity of tail, or gonad	Tissues other than those injected with RNA exhibited RNAi, indicating that RNAi is systemic. Also, injection of adults sometimes led to offspring with RNAi, indicating that transgenerational inheritance of RNAi is possible. These results suggested that the RNAi mechanism existed throughout the whole organism.

(b)		
Target of specificity	Range tested	Result
Gene regions	One exon and multiple exons, intron, or promoter	RNAi occurred only when the coding sequence of the mRNA was targeted, indicating that RNAi works through posttranscriptional regulation.
Conserved gene region		RNAi led to an unexpected phenotype, indicating that RNAi affects genes with a similar sequence to the gene of interest.
Gene of interest	*unc-22*, *unc-54*, *fem-1*, *hlh-1*, *gfp*, or *mex-3*	The target genes for RNAi were nonessential and had previously been characterized with easily identifiable visual phenotypes. Also, the relationship between the gene's expression and phenotype was in the manipulable direction for RNAi knockdown (i.e. reduced expression increased the severity of the phenotype).
Transgenic line expressing 2 GFP reporter proteins		RNAi occurred in individual cells of the organism.
*mex-3* in an in situ hybridization experiment		The target of RNAi was a gene that is abundant in early embryos (a useful developmental period for an in situ experiment). Endogenous mRNA disappeared suggesting it was destroyed, visually indicating that mRNA (not precursor mRNA, nor protein) was the target of RNAi.

Experiments that (a) identified the triggers in the RNAi mechanism and (b) identified the target of the specificity in the RNAi mechanism.

For example, in the late 1990s, dsRNA was found to be causally specific for the RNAi mechanism (Table 2). The dsRNA was investigated due to it being accidently produced in earlier experiments, as it was found that:

… polymerases, although highly specific, produce some random or ectopic transcripts. DNA transgene arrays also produce a fraction of aberrant RNA products^3^… we surmised that the interfering RNA populations might include some molecules with double-stranded character. (Fire *et al*. 1998, p. 807)

Fire *et al*. (1998) tested the specificity of RNA molecules to control the RNAi mechanism in *C. elegans* (Table 3a). The dsRNA was identified as the cause of sequence-specific regulation of mRNA, as they:

… investigate[d] the requirements for structure and delivery of the interfering RNA. To our surprise, we found that double-stranded RNA was substantially more effective at producing interference than was either strand individually. Fire et al. (1998, p. 806)

Therefore, the study was a conclusive demonstration of how dsRNA can be used to control the RNAi mechanism.

After dsRNA was identified as a trigger, biologists wondered how it could bind and sequence specifically cleave mRNA. They found that dsRNA is processed into small RNA fragments (antisense and sense) in multiple organisms and suggested that these were necessary for RNAi (Hamilton and Baulcombe 1999; Hammond *et al*. 2000; Parrish *et al*. 2000; Zamore *et al*. 2000). These small interfering RNAs (siRNAs), 21–23 nucleotides in length, were shown to sequence specifically guide the cleavage of the mRNA (Elbashir *et al*. 2001b) (Table 2).

Two years after the RNAi technique was developed, the endogenous effector component that degrades the target mRNA was identified as the RISC (Table 2). The endonuclease that cuts the target mRNA sequence specifically was identified in *Drosophila melanogaster* cells as Argonaute, which is part of the RISC (Hammond *et al*. 2000; Martinez *et al*. 2002). The effector that cleaves dsRNA into siRNAs was identified as a ribonuclease type III named Dicer (Bernstein *et al*. 2001). Biologists then pursued further mechanistic details, such as the functions of different forms of the Argonaute protein (Rana 2007).

### The third phase: application of the trigger(s) as a technique

In the third phase of technique development, biologists conclusively determine that when the trigger is introduced into the experimental system, it achieves some intended effect on the target of the specificity. The trigger is exploited in 3 types of investigative strategies: to intervene on a cellular experimental system (for example, RNAi); to manipulate an effector's activity in a noncellular experimental system (for example, restriction enzymes); or as a tracer, to follow a biological process (see Griesemer 2007; for example, fluorescent proteins) (Table 1). At this stage, a deep understanding of the mechanism underlying the technique is not necessary for the technique to work.

For example, the RNAi technique was first applied in the Fire *et al*.’s (1998) paper “Potent and specific genetic interference by dsRNA in *Caenorhabditis elegans*” (Table 2). The study was a conclusive demonstration of how dsRNA can be applied as a molecular technique to manipulate gene expression in *C. elegans*. Fire *et al*. (1998, p. 810) concluded that RNAi:

… adds to the tools available for studying gene function in *C. elegans*. In particular, it should now be possible functionally to analyse many interesting coding regions^21^ for which no specific function has been defined.

Interestingly, Fire *et al*. (1998, p. 810) explicitly stated that they did not understand the biological function of the RNAi mechanism:

Whatever their target, the mechanisms underlying RNA interference probably exist for a biological purpose.

It is important to note that when a molecular technique is developed for an organismal experimental context (Table 1), it is typically tested in a genetic model organism system. For example, RNAi was first developed using the model organism *C*. *elegans* (Fire *et al*. 1998). A model organism provides standardized experimental systems that are relatively well characterized at the molecular level, which therefore act as a prototype for technique development (Ankeny 2000; Leonelli and Ankeny 2013). When a technique has been validated in a model organism, there is the expectation that due to the fundamental unity of living systems, the technique will be able to be applied to other organisms. The use of model organisms in this phase is particularly important given the complexity and cost of molecular experiments.

### The fourth phase: maturation of the technique

In the fourth phase of technique development, the technique has been established, and biologists improve and expand its performance. The scientific community invests considerable research activity into characterizing, both spatially and temporally, the mechanism in natural systems. Therefore, the technique generates further research on the biological mechanism that underlies it. The new knowledge acquired may improve access to the mechanism or allow the technique to be better controlled, enabling the technique to continue to be refined and standardized.

Immediately following the seminal RNAi study of Fire *et al*. (1998), the technique was shown to work in multiple organisms (Table 2): *C. elegans* (Fitzgerald and Schwarzbauer 1998; Montgomery *et al*. 1998; Ogg and Ruvkun 1998; Page and Winter 1998; Skop and White 1998; Tabuse *et al*. 1998; Timmons and Fire 1998); 2 species of plants, *Nicotiana tabaccum* and *Oryza sativa* (Waterhouse *et al*. 1998); and *D. melanogaster* (Kennerdell and Carthew 1998). In mammals, RNAi using dsRNA initially failed due to the immune response elicited; however, when siRNAs were used, gene expression could be altered (Elbashir *et al*. 2001a). RNAi has become a highly selective molecular technique for reducing expression of a target gene, and today it is widely used for both fundamental and applied research (Mello and Conte 2004; Deng *et al*. 2014; Fellmann and Lowe 2014). To this day, the biological mechanism of RNAi is still being investigated.

### Molecular technique development

The 4 phases I have identified are necessary features of technique development when derived from a natural system. I have shown that 8 highly successful molecular techniques have these 4 phases of development (Table 2). Additional techniques that likely follow this phased development from natural systems, include reverse transcription, transposable elements, molecular cloning (utilizing a plasmid vector), monoclonal antibodies, site directed mutagenesis, recombinases, optogenetics, and immunotherapy (utilizing endogenous immune system components).

The development of new molecular techniques helps accelerate research in genetics and generates new scientific knowledge that would otherwise not exist. A new technique can also help uncover previously undetected biological phenomena, in turn leading to the development of yet another technique. For example, restriction enzymes were instrumental to the initial detection of the RNAi phenomena (Napoli *et al*. 1990; van der Krol *et al*. 1990), and during the application phase of development for RNAi, green fluorescent protein was used to visualize that the RNAi mechanism occurs within cells (Table 3b; Fire *et al*. 1998). Therefore, the molecular techniques used in genetics build upon one another and are cumulative.

### Scientific values and the success of biological techniques

Three scientific values (Kuhn 1977; Darden 1991; Douglas 2013) are important for the genetics community's adoption of a molecular technique. First, a technique should be fruitful for further research. Techniques generate knowledge and open up new areas of research. For example, RNAi has helped geneticists manipulate RNA thus leading to a more sophisticated understanding of the function of RNA (Mello and Conte 2004) and has allowed geneticists to manipulate genes that are lethal in development in order to investigate their functions (for example, Fitzgerald and Schwarzbauer 1998). Second, a technique should allow expansion of its scope of application far beyond its original biological context. A technique that has applications in many experimental contexts means that a larger scientific community can use the technique. In addition, a technique that can be used in mammals is particularly desired due to the value placed on medical and therapeutic research. For example, the RNAi effector, RISC, is present in all eukaryotes (Cerutti and Casas-Mollano 2006) and RNAi can be used in human cell lines (Elbashir *et al*. 2001a). Third, a technique should have “extendability.” A technique should accommodate modifications so that it can be used for different capabilities to its original purpose. A technique can therefore become the progenitor for a family of related techniques. For example, a form of RNAi has been developed that used RNA molecules targeted at promoters to increase rather than decrease gene expression (Li *et al*. 2006). These 3 scientific values have helped establish the success of the 8 biological techniques in the scientific community.

## Concluding remarks

I have highlighted 8 highly successful techniques of contemporary genetics that are derived from natural systems. The history of these techniques, I have shown, falls into 4 distinctive phases. It is an open question whether genetics will continue to progress through the development of molecular techniques derived from natural systems. Perhaps knowledge construction in biology requires a natural systems strategy. Alternatively, there is some evidence that geneticists working on synthetic biology have started to use rational design in organisms (Hutchison *et al*. 2016); for example, the high profile “Human Genome Project–Write” aims to artificially synthesize the whole human genome to improve medical research and therapeutics (Boeke *et al*. 2016). However, geneticists often find that rational design is laborious and that selection methods on natural systems lead to improved technique development and outcomes (Silverman 2003). Furthermore, a rational design strategy cannot be used to access the causal structure of molecular mechanisms when no comprehensive understanding of these mechanisms exists.

Genetics has a historically accumulated set of molecular techniques to manipulate, intervene on, and trace molecular processes. Progress in genetics is greatly dependent on its powerful techniques—the cycle between discovery of biological phenomenon, mechanistic understanding, and application as a technique will continue.

## Data Availability

The author affirms that all data necessary for confirming the conclusions of the article are present within the article and tables.
